# Glass ionomer open exposure and closed exposure of palatally displaced canines: a randomised controlled trial comparing postoperative pain perception and complications

**DOI:** 10.1093/ejo/cjag011

**Published:** 2026-03-17

**Authors:** Anna Dahlén, Viktoria Tagesson, Damon Taheri, Ken Hansen, Larisa Krekmanova, Julia Naoumova

**Affiliations:** Department of Orthodontics, Institute of Odontology, Sahlgrenska Academy, University of Gothenburg, Box 450, 405 30 Gothenburg, Sweden; Public Dental Service, University Clinic of Orthodontics, 413 90 Gothenburg, Region Västra Götaland, Sweden; Public Dental Service, Clinic of Orthodontics, 531 60 Lidköping, Region Västra Götaland, Sweden; Private Practice, 20 Haven Road, Canford Cliffs, Poole BH13 7LP, United Kingdom; Public Dental Service, University Clinic of Orthodontics, 413 90 Gothenburg, Region Västra Götaland, Sweden; Department of Paediatric Dentistry, Institute of Odontology, Sahlgrenska Academy, University of Gothenburg, Box 450, 405 30 Gothenburg, Sweden; Department of Orthodontics, Institute of Odontology, Sahlgrenska Academy, University of Gothenburg, Box 450, 405 30 Gothenburg, Sweden; Public Dental Service, University Clinic of Orthodontics, 413 90 Gothenburg, Region Västra Götaland, Sweden

**Keywords:** surgical exposure, cuspid, ectopic, ectopic eruption, impacted maxillary canines, orthodontic, patient-reported outcome measures, surgery, tooth eruption

## Abstract

**Background:**

The most common surgical interventions for palatally displaced canines (PDCs) are open and closed exposure, yet there is no consensus on which exposure technique is preferable.

**Objectives:**

To compare glass ionomer open exposure (GOPEX) with closed exposure (CE) in terms of patient-reported outcomes, surgical duration, and complications.

**Trial design:**

The trial design was a single centre, randomised controlled trial, with a 1:1 allocation of two parallel groups.

**Methods:**

Patients aged ≤18 years, with a unilateral PDC in sector 2–5, requiring surgical exposure were randomly allocated to GOPEX or CE. Patient-reported outcomes were collected through a questionnaire completed over the phone daily from the day of exposure until symptom resolution. Surgery duration, recorded by the operator, and complications within four weeks, was obtained from the patient’s record. The outcome assessor and the individual conducting the follow-up calls were blinded to group allocation. Repeated measures analyses were used for data collected at multiple time points. Mann–Whitney U-test, chi-square test or Fisher’s exact test, as appropriate was used for other between-group comparisons.

**Results:**

Of the 92 patients randomised, 83 completed the intervention: 43 in the GOPEX group (13.8 ± 1.5 years) and 40 in the CE group (13.6 ± 1.2 years). Throughout the full postoperative period, the groups did not differ significantly in pain levels, percentage of pain-free patients, analgesic use, or chewing difficulty. During the first postoperative week, however, the GOPEX group reported higher pain (fold change 1.78; 95% CI: 1.07–2.99; *P* = 0.028). Fewer GOPEX patients were pain-free at one week, and they consumed more analgesics on postoperative days 4 and 5. Surgery duration was similar between groups, and complications were rare and comparable.

**Conclusions:**

Although the GOPEX group reported more pain during the first postoperative week, overall pain scores and analgesic consumption across the full postoperative period did not differ between groups. No significant differences were found in surgery duration or complications.

**Trial registration:**

www.researchweb.org/is/sverige, registration number: 279469.

## Introduction

The prevalence of palatally impacted canines varies between 1.8% and 2.2% [1, 2]. Failure of spontaneous canine eruption is more common in girls than in boys [3, 4]. In most cases, the canines are palatally positioned [4, 5]. Extraction of the primary canine to facilitate the eruption of the palatally displaced canine (PDC) is an effective interceptive treatment [6, 7]. However, if this approach is unsuccessful, if the canine is impacted, or if signs of root resorption of adjacent teeth are detected, surgical exposure of the PDC is recommended [8]. Two different methods have been developed for exposure of a PDC, open and closed surgical exposure. In open exposure, the overlying bone and mucosa are excised, and the exposed canine is covered with a dressing or packing, which may vary depending on the surgeon’s preferences [9]. In closed exposure (CE), a flap is raised, and an attachment is bonded to the PDC before the flap is sutured back into its original position. Orthodontic traction is then required to guide the tooth from beneath the mucosa and is typically initiated shortly after surgical exposure [8, 10]. A modified open exposure technique has been used in Gothenburg for more than 40 years [11, 12]. Instead of surgical packing, Glass Ionomer Cement (GIC) is applied to the canine. Due to its strong adhesion to enamel, the glass ionomer does not detach within a few weeks but can remain on the tooth until it has spontaneously erupted above the gingiva. This technique is referred to as glass ionomer open exposure (GOPEX) [13, 14].

Previous studies comparing open and CE indicate similar outcomes regarding postoperative pain perception and symptoms such as difficulties with oral hygiene, eating, and speaking [15, 16] or increased pain and impairment after open exposure [17-19]. Open exposure has been reported to both have shorter [16, 19], and longer surgery duration [17] or no significant differences between the open and closed techniques [15, 18]. Also, complications after surgery have conflicting results and been reported to be more common after open exposure [19], similar between open and CE [15], or with more severe complications after open and more minor complications after CE [18]. These inconsistencies in the existing literature may contribute to the fact that the clinician’s personal preferences often determine which technique will be used and that orthodontists hold widely differing views regarding the advantages and disadvantages, both with respect to postexposure outcomes and other important factors such as the duration of orthodontic treatment and periodontal status after orthodontic treatment [13]. Both a systematic review by Sampaziotis *et al*. [20] and a Cochrane review by Parkin *et al*. [21] on surgical exposure of maxillary canines highlighted the need for additional high-quality randomised clinical trials. Furthermore, all previous studies, except for one [18], have used surgical packing rather than GIC in open exposure, and no RCT has collected daily data until patients were fully symptom-free, resulting in insufficient information on patient perceptions to compare GOPEX and CE.

Therefore, the primary aim was to compare GOPEX and CE of PDCs postoperatively in terms of patients’ perceptions of pain. Secondary aims were to compare bleeding and swelling, analgesic use, impact on daily activities, surgery duration, and surgical complications other than pain.

The null hypothesis was that there were no significant differences between the groups for any of the parameters analysed.

## Materials and methods

### Ethical issue

The study protocol (reg.no. 037-17) was approved by the Regional Ethics Review Board at the University of Gothenburg, Sweden. Before participation in the study, both patients and their legal guardians received oral and written information, and informed consent was provided by the legal guardian, in accordance with the Declaration of Helsinki.

### Registration

The trial was registered in ‘FoU i Sverige’ (R&D in Sweden, project database) (https://www.researchweb.org/is/sverige), registration number: 279469.

### Trial design and randomisation

This was a single-centre, two-arm, parallel group, randomised controlled clinical trial with a 1:1 allocation ratio.

Patients were randomly allocated to one of the two surgical exposure techniques (GOPEX versus CE) in blocks of variable sizes (2, 8, and 10), with stratification performed for the surgeon and the treating orthodontist. An online-generated randomisation list was created by one of the authors (JN) using https://www.randomizer.org/. The allocation concealment was stored securely at Clinic of Orthodontics, University Dental Care, Gothenburg, Sweden, and was not available to the recruiting orthodontists (VT, AD). Allocation concealment was held by one individual (DT), who was not involved in recruiting or treating the patients, and who was contacted by the treating clinician at the time for allocation, i.e. when written consent was obtained.

### Subjects, eligibility criteria and setting

The trial took place at the University Clinic of Orthodontics and Paediatric dentistry, Public Dental Service, Region Västra Götaland, Gothenburg, Sweden. Children and adolescents with a PDC were identified by a general dental practitioner at one of 15 public dental clinics in Gothenburg, between March 2017 and April 2024. They were referred to the Orthodontic Clinic, where the eligible candidates for the trial were consecutively invited to participate in the study.

#### Inclusion criteria

- Unilateral PDC
- Planned for surgical exposure and orthodontic treatment without the need for extraction of permanent teeth
- Canine positioned in sector 2–5 according to Ericsson and Kurol [7] (panoramic radiographs)
- Age ≤18 years at the time of surgical exposure
- With or without persisting maxillary primary canine at the time of exposure

#### Exclusion criteria

- Need for general anaesthesia
- Craniofacial syndromes including cleft lip and/or palate

### Interventions

All included patients were referred to a paediatric dental specialist, as surgical procedures for this age group are managed by them in accordance with county regulations from Region Västra Götaland. A detailed description of the surgical procedure to standardise the surgical exposures was included in the referral. Three paediatric dentistry specialists with ≥10 years of experience of both surgical techniques performed all the exposures according to the standardised protocol. All surgical exposures were performed in the morning, except for four procedures, which were carried out at 1:00 p.m.

#### Sedatives/anxiolytics

Sedative medication with midazolam 1 mg/mL, Diazepam and/or nitrous oxide was offered preoperatively to the patients and the dose was adjusted according to the patient’s weight. Paracetamol 500–2000 mg, depending on weight, were given to all patients preoperatively at the clinic, in accordance with guidelines set by the International Association for the Study of Pain [22, 23].

#### Local anaesthetics

Prior to the operation, the surgical area was anaesthetised with topical Lidocaine gel 5% for 3–5 min followed by buccal, intrapapillary and palatal infiltration anaesthesia (Xylocaine Dental Adrenalin 20 mg/mL + 12.5 µg/mL).

#### Operative procedure

If the deciduous canine was present, it was extracted before the exposure. A mucoperiosteal flap was raised from the gingival sulcus of the premolars to the contralateral incisor to locate the PDC. The PDC was then freed from the follicle and bone was removed to expose the crown with a noncrosscut carbide bur. The surgical area was continuously flushed with physiological saline.

The surgical exposure then continued as described below depending on allocation.

## GOPEX

**Figure 1:**
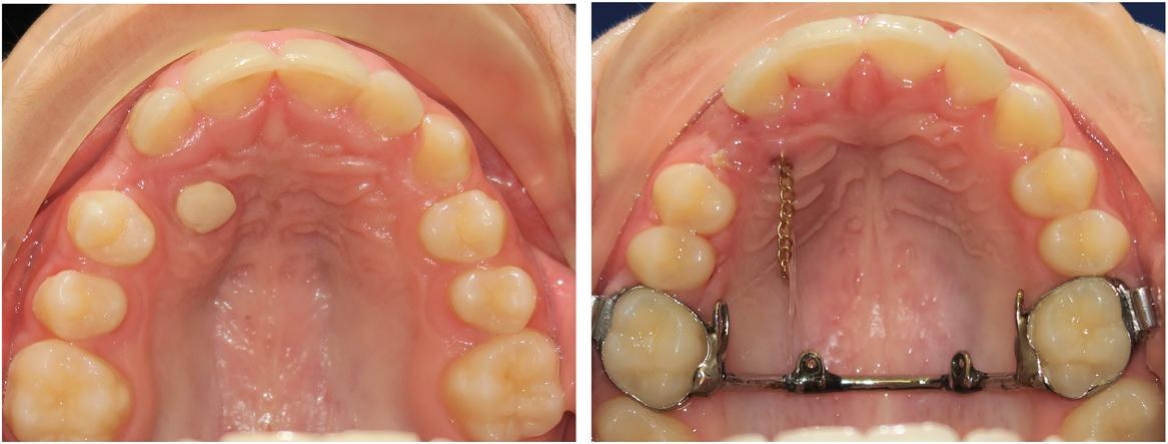
Left picture shows 13 exposed with glass ionomer open exposure (GOPEX), while the right picture shows 13 exposed with closed exposure (CE).

A 6 mm biopsy punch was used to make an opening through the mucoperiosteal flap at the location of the cusp tip of the PDC before the flap was sutured back in its original position. Haemostasis was achieved by applying compression with a mirror and a haemostatic agent (Xylocaine Dental Adrenaline or Cyklokapron 5%) when needed. The operation area was dried before the glass ionomer cement (KetacFil 3M ESPE or GC Fuji IX) was applied to the cusp tip of the exposed PDC (Fig. 1).

## CE

After achieving a dry tooth surface, the enamel on the crown tip was prepared (35% phosphoric acid, Adhese Universal VivaPen) and an eyelet was bonded with a light cured flowable composite (Transbond™XT, 3M Unitek). The mucoperiosteal flap was then perforated with a 2 mm biopsy punch, making a hole large enough for a chain attached to an eyelet to pass through. The chain was stretched and bonded to the buccal surface of an adjacent tooth. The surgical site was flushed with physiological saline before the flap was sutured back in its original position (Fig. 1).

After the operation, all patients were recommended paracetamol and ibuprofen for up to 5 days, with the dosage adjusted according to weight. Patients were advised to eat soft food and to avoid brushing or brush very gently in the area of exposure for 3–5 days.

### Canine position

**Figure 2:**
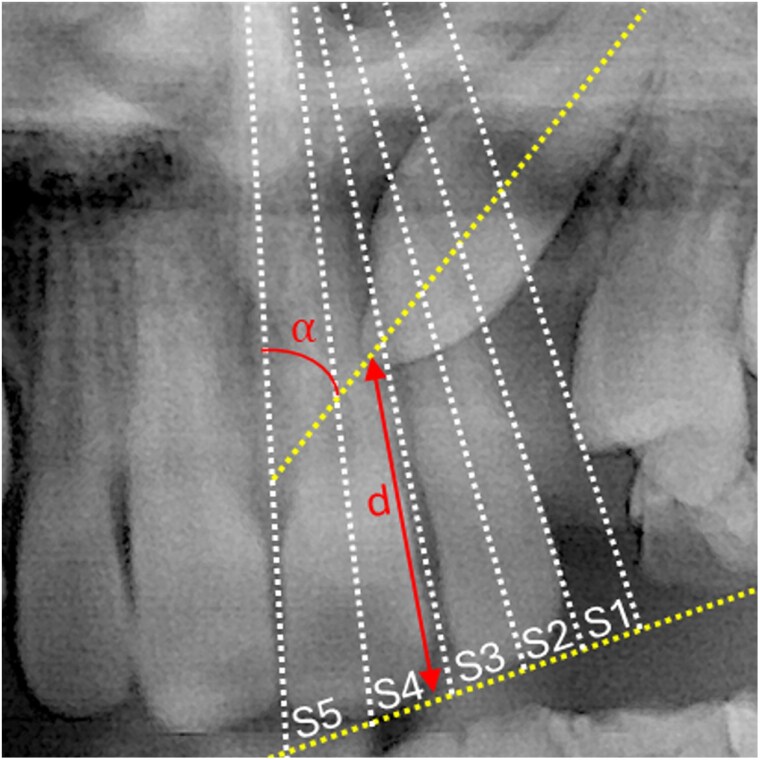
Panoramic measurements using the method described by Ericson and Kurol [7]. α: angle, angle formed by the long axis of the canine and the midline; d-distance, distance in mm from the canine cusp tip to the occlusal plane, and sector: mesiodistal crown position in sector 1–5.

The position of the PDC was assessed by a single-blinded assessor (DT) from pretreatment panoramic radiographs, as described in previous studies [7] using Facad Software (version 3. 14.1.1111, Ilexis AB, Linköping, Sweden) (Fig. 2). To assess intra-examiner reliability, 20 patients were randomly selected and remeasured.

- Alpha angle: the angle between the long axis of the canine and the midline. The midline was defined as a line going from the spina nasalis anterior and between the central incisors.
- D-distance: the distance in millimetres from the canine cusp tip to the occlusal plane. The occlusal plane was defined as a line from the distal cusp tip of the first molar to the incisal edge of the central incisor on the same side as the PDC.
- Sector: mesiodistal crown position of the PDC in sector 2–5.

### Outcome measurements

#### Patient-reported outcome

Pre-surgery, at the same appointment where patients gave their consent to participate, they also completed the Dental Subscale of the Children’s Fear Survey Schedule (CFSS-DS) [24] (Supplementary Material S1). Each question in the CFSS-DS scores 1–5, from ‘not at all afraid’ to ‘Extremely afraid’, with a total score range of 15–75. A score of 38 or more has been associated with clinical dental fear and the survey has been widely used and validated for internal consistency, validity and reliability in different populations including in Sweden [25, 26].

After surgery, patients were contacted daily by telephone, starting in the evening of the day of surgery (day 1) and continuing until they reported being pain-free, symptom-free, and no longer using analgesics. ‘Pain-free’ was defined as reporting ‘no pain at all’ (pain score 0) on the pain scale explained below. ‘Symptom-free’ was defined as reporting ‘no pain at all’ along with no bleeding, swelling, or jaw function issues related to the exposure. The author making the calls read the questions aloud to the patients. If patients had difficulty understanding any questions or terms, the author provided clarification. The phone interviews took about 5–7 minutes. The questionnaire (Supplementary Material S2) included questions about whether the patient experienced any part of the exposure procedure as particularly unpleasant, and if so, what part. Questions regarding pain addressed both the time of day it occurred and its characteristics. A Numerical Rating Scale (NRS) ranging from 0 to 10, where 0 indicated ‘no pain at all’ and 10 indicated the ‘worst pain imaginable’ was used to assess pain intensity. Further questions addressed analgesic use, bleeding, swelling, and the impact on school and leisure activities. A previously validated questionnaire on jaw function impairment [27], proven to be reliable and to possess sufficient internal consistency, was also included, partly rephrased to fit the study’s methodology.

#### Surgery duration

Surgery duration was measured in minutes from the first incision to the last suture, including the time to stop any bleeding to ensure that the surgical field was dry before applying the glass ionomer or bonding the eyelet. Anaesthesia time was not included.

#### Complications

Postoperative complications were recorded for up to four weeks following surgery and collected from patients’ dental records. A complication was defined as any visit to the dentist, initiated by the patient or legal guardian that resulted in the dentist carrying out an intervention or procedure. Loss of GIC was also considered a complication, even if it did not result in a dental visit or an intervention. Complications were categorized as major or minor. Major complications included bleeding, swelling, the need for second surgery and infection. Infection was defined as requiring a prescription of antibiotics. Minor complications included debonding of the GIC that did not require intervention, or detachment of the chain from a tooth other than the PDC.

### Blinding

Because of the study design, it was not feasible to blind the patient or the paediatric dentist to the allocation group. However, the outcome assessor (before and after treatment) and the individual contacting the patients after the surgery were blinded to the allocation group and not involved in the treatment.

### Sample size calculation

A difference of 20 mm on the VAS pain scale (equivalent to an NRS difference of 2) has been reported as clinically relevant [28]. The standard deviation of pain after surgical exposure of PDC has previously been reported as 2.2 on a 10-point Likert scale [15]. Based on these data, the sample size for the present study was calculated under the assumption of a mean NRS difference of 2 between groups, with a standard deviation of 2.2. Using a type I error of 5% and a type II error of 20%, it was estimated that 40 patients per group would be required. The target sample size was increased to 92 participants to compensate for anticipated dropouts.

### Statistical analysis

The statistical analysis was performed with IBM SPSS Statistics for Windows (Version 28.0. Armonk, NY: IBM Corp. Released 2021) for all analysis except repeated measures analyses for which SAS (version 9.4 M9, SAS Institute, Cary, NC, USA) was used. The level of statistical significance was set to 0.05, and all tests were two-sided.

Descriptive statistics, including the arithmetic mean were calculated for the baseline variables and were stratified by treatment group.

For pain intensity (NRS), both mean and median values were calculated. Group comparisons were performed using the Mann–Whitney U-test for continuous variables, and Chi-square test or Fisher’s exact test (when expected cell counts were <5) for categorical variables.

Pain scores were analysed using a repeated-measures linear mixed model. To improve normality and stabilise variance, pain scores were log-transformed before analysis. The model included operation, day (≤7 and ≤14 postoperative days), their interaction, and surgeon as fixed effects. Days 15–20 were excluded due to minimal variance when only two patients per group reported pain. A repeated-measures structure with day within patient and an unstructured covariance matrix accounted for within-subject correlation. Model results were back-transformed, yielding least-squares geometric mean pain scores and fold changes (geometric mean ratios) with 95% confidence intervals, based on operation-by-day and operation main effects with pairwise comparisons from the fitted model.

Binary outcomes (pain free percentage, analgesics and difficulties chewing hard food) were analysed using generalised estimating equations (GEE) with a binomial distribution and logit link. The model included the same fixed effects except surgeon and assumed an exchangeable working correlation to account for within-subject correlation across days. Predicted proportions (multiplied with 100 to convert to percentage) were obtained via the inverse logit transformation, and absolute differences in percentage with 95% confidence intervals were derived from model-based least-squares means and pairwise contrasts. For other jaw function outcomes, the GEE model could not be estimated due to limited variability (few patients reported difficulties). Consequently, only descriptive statistics (*n*, %, ‘not applicable’) were presented for these outcomes, as well as for questions regarding school, leisure activities, disturbed sleep, bleeding, and swelling.

For the purposes of analysis, responses to question 6 and 7 were dichotomized into two groups: ‘not at all’ and ‘slightly difficult’ versus ‘very difficult’ and ‘extremely difficult’. As the reason for responding ‘not applicable’ was unknown, these responses were treated as missing in the analysis and were not included in the percentage calculations in the descriptive table.

Intra-examiner reliability, assessed using Cohen’s weighted kappa for categorical data and intraclass correlation for ordinal measures, showed excellent agreement (sector 0.93, alpha angle 0.99, d-distance 0.99).

## Result

### Demographics

**Figure 3:**
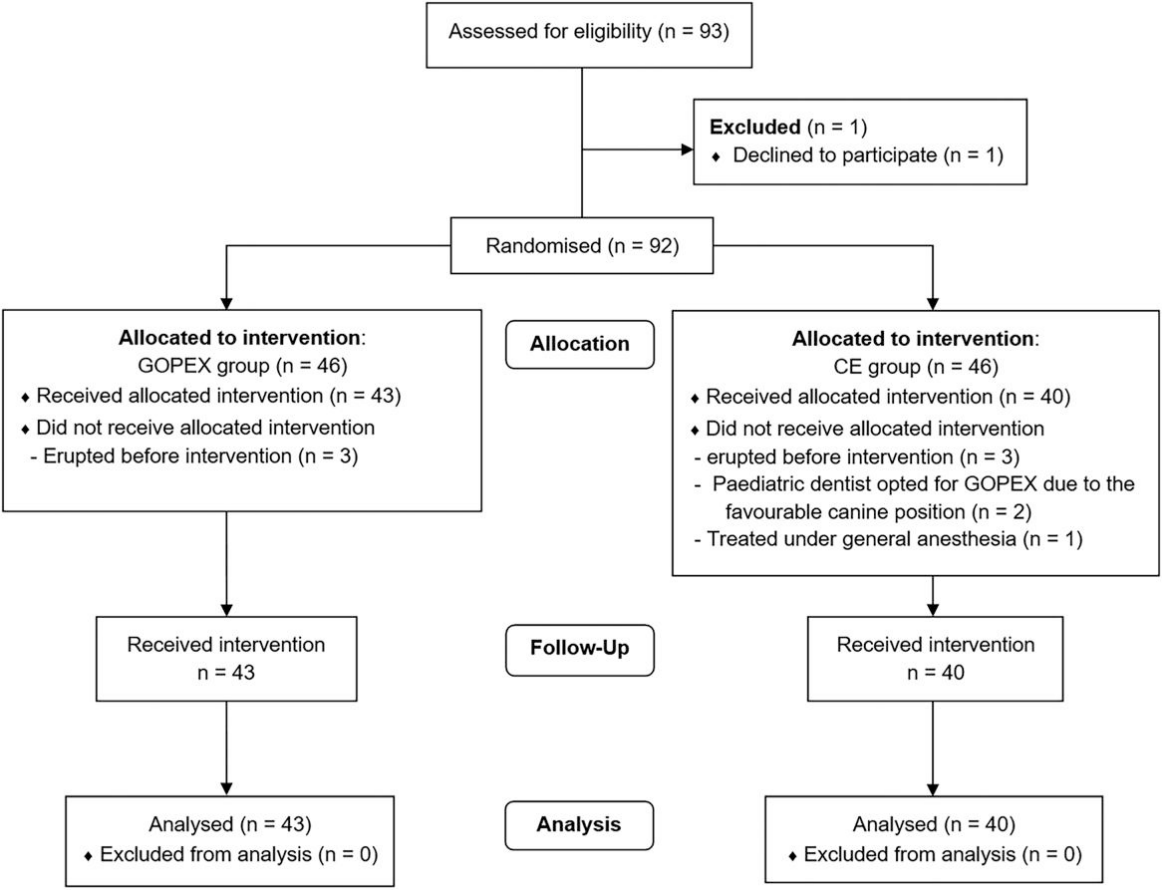
Consort flow chart.

A total of 93 patients were invited to take part in the study. One patient declined to participate. Ninety-two patients were randomised, and 83 patients received the allocated intervention, resulting in 43 patients in the GOPEX group and 40 patients in the CE group (Fig. 3).

The patients and the pretreatment characteristics are described in Table 1. The GOPEX group had more PDCs in sector 2 and 4, whereas the CE group had more PDCs in sector 3 and 5. In one patient, the lateral incisor was missing, but the PDC was located in sector 5, overlapping the central incisor. There were no significant differences between the two intervention groups in terms of sex, age, impaction side or severity of canine impaction (Table 1). The median dental fear score in the GOPEX group was 23.0 (IQR: 20.0–32.0) and in the CE group 21.0 (IQR: 18.0–27.0) indicated slightly higher dental fear score in the GOPEX group compared with the CE group, but the difference was not statistically significant (*P* = 0.070).

**Table 1:** Sample characteristics.

		GOPEX group (*n* = 43)			CE group (*n* = 40)	
	*n*	Mean age (years)	SD	*n*	Mean age (years)	SD
Female	27	13.8	1.5	22	13.2	1.3
Male	16	13.7	1.4	18	14.0	1.1
Total	43	13.8	1.5	40	13.6	1.2
Tooth						
	***n***	**%**		***n***	**%**	
13	19	52.8		17	47.2	
23	24	51.1		23	48.9	
Canine position^a^						
Sector 2	8	18.6		3	7.5	
Sector 3	12	27.9		18	45.0	
Sector 4	16	37.2		10	25.0	
Sector 5	7	16.3		9	22.5	

		Mean	SD		Mean	SD
Alpha angle (°)		30.0	10.9		33.9	13.5
D-distance (mm)		11.8	2.2		11.7	2.5

CE, closed exposure; GOPEX, glass ionomer open exposure; *n*, number of patients; SD, standard deviation.

^a^Canine position according to Ericson and Kurol [7].

### Patient-reported outcome

All patients who underwent the allocated intervention responded to the CFSS-DS and participated in the telephone interviews.

#### Exposure experience

Ten patients (23%) in the GOPEX group and twelve patients (30%) in the CE group reported experiencing certain parts of the procedure as particularly unpleasant, with anaesthesia being the most common issue in both groups (64%), followed by bone removal (9%).

#### Pain intensity and characteristics

**Figure 4:**
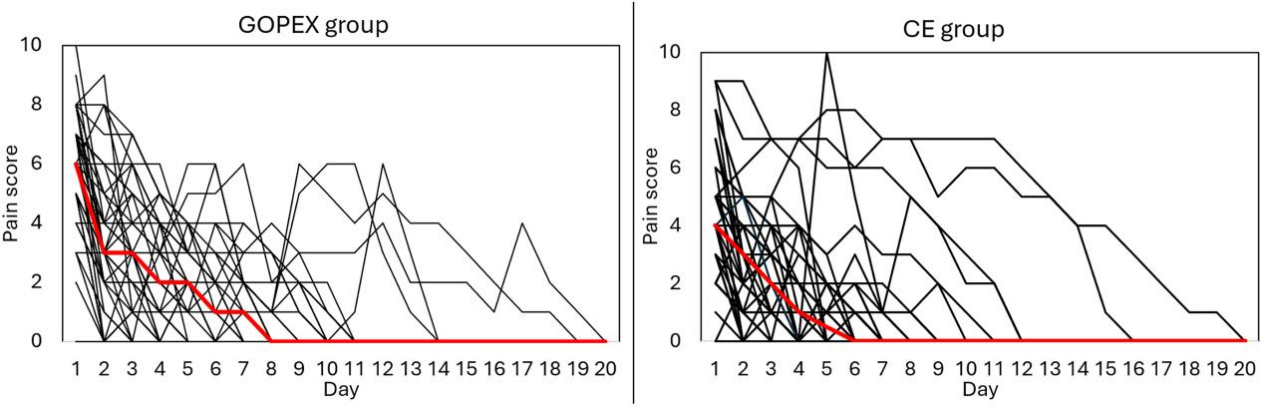
Spaghetti plots illustrating reported pain in the glass ionomer open exposure (GOPEX) group (left) and the closed exposure (CE) group (right), with red lines denoting median pain scores.

The mean and median pain scores from the evening of exposure (day 1) to day 20, when all patients were pain-free, are shown in Table 2. Spaghetti plots (Fig. 4) illustrate a similar decline in pain for both groups, with considerable individual variation within each group.

**Table 2:** Pain score (0–10) for days 1–20 for glass ionomer open exposure (GOPEX) and closed exposure (CE) groups.

Day	GOPEX group (*n* = 43)			CE group (*n* = 40)		
	Mean (SD)	Median (IQR)	Min-Max	Mean (SD)	Median (IQR)	Min-Max
1	6.00 (2.22)	6.0 (5.0–8.0)	0–10	4.50 (2.35)	4.0 (3.0–6.0)	0–9
2	3.44 (2.43)	3.0 (2.0–5.0)	0–9	2.80 (2.16)	3.0 (1.0–4.0)	0–9
3	2.98 (2.19)	3.0 (1.0–5.0)	0–7	2.35 (2.03)	2.0 (1.0–3.8)	0–7
4	2.21 (1.92)	2.0 (0.0–4.0)	0–6	1.65 (2.18)	1.0 (0.0–2.8)	0–7
5	1.77 (1.69)	2.0 (0.0–3.0)	0–6	1.51 (2.39)	0.5 (0.0–2.0)	0–10
6	1.47 (1.73)	1.0 (0.0–2.0)	0–6	1.15 (1.99)	0.0 (0.0–1.8)	0–8
7	1.14 (1.42)	1.0 (0.0–2.0)	0–6	0.75 (1.82)	0.0 (0.0–0.8)	0–7
8	0.65 (1.11)	0.0 (0.0–1.0)	0–4	0.75 (1.89)	0.0 (0.0–0.0)	0–7
9	0.70 (1.39)	0.0 (0.0–1.0)	0–6	0.60 (1.60)	0.0 (0.0–0.0)	0–7
10	0.44 (1.28)	0.0 (0.0–0.0)	0–6	0.48 (1.52)	0.0 (0.0–0.0)	0–7
11	0.33 (1.17)	0.0 (0.0–0.0)	0–6	0.43 (1.48)	0.0 (0.0–0.0)	0–7
12	0.42 (1.14)	0.0 (0.0–0.0)	0–6	0.28 (1.22)	0.0 (0.0–0.0)	0–6
13	0.23 (0.81)	0.0 (0.0–0.0)	0–4	0.25 (1.10)	0.0 (0.0–0.0)	0–5
14	0.14 (0.68)	0.0 (0.0–0.0)	0–4	0.20 (0.88)	0.0 (0.0–0.0)	0–4
15	0.12 (0.54)	0.0 (0.0–0.0)	0–3	0.13 (0.65)	0.0 (0.0–0.0)	0–4
16	0.07 (0.34)	0.0 (0.0–0.0)	0–2	0.08 (0.47)	0.0 (0.0–0.0)	0–3
17	0.12 (0.63)	0.0 (0.0–0.0)	0–4	0.05 (0.32)	0.0 (0.0–0.0)	0–2
18	0.07 (0.34)	0.0 (0.0–0.0)	0–2	0.03 (0.16)	0.0 (0.0–0.0)	0–1
19	0.02 (0.15)	0.0 (0.0–0.0)	0–1	0.03 (0.16)	0.0 (0.0–0.0)	0–1
20	0.00	0.0 (0.0–0.0)	0–0	0.00	0.0 (0.0–0.0)	0–0

Pain score assessed on a numerical rating scale (NRS) from 0 to 10.

IQR, interquartile range; Max, maximum; Min, minimum; SD, standard deviation.

**Figure 5:**
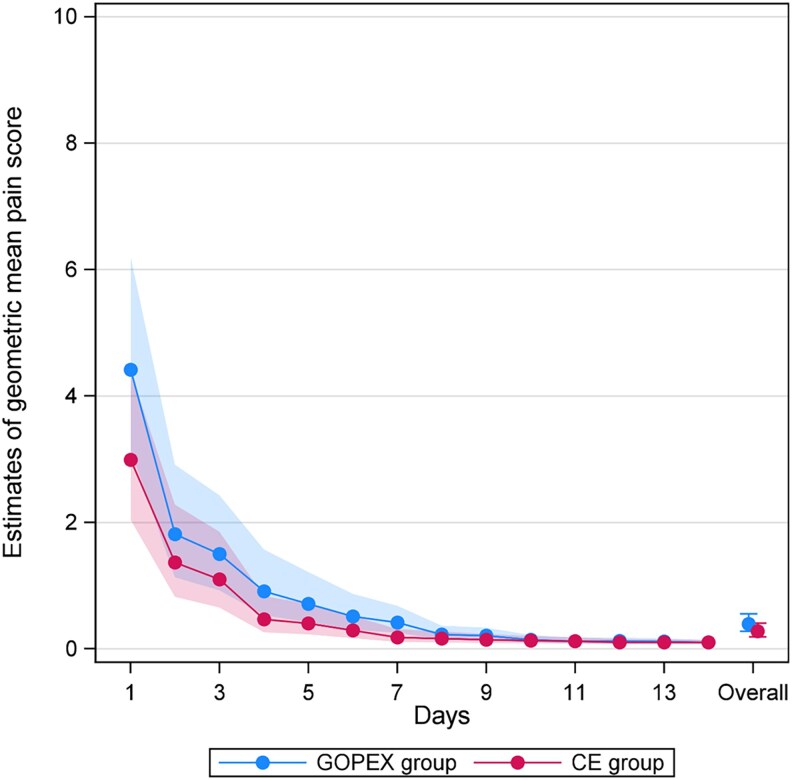
Geometric mean pain scores and corresponding 95% confidence intervals for days 1–14, stratified by treatment arm: glass ionomer open exposure (GOPEX) and closed exposure (CE).

During the first 14 postoperative days, the overall fold change in geometric mean pain scores—representing the ratio between groups (GOPEX vs CE)—was 1.41 (95% CI: 0.93–2.15; *P* = 0.103). As pain scores in both groups were low after day 7, the overall 14-day comparison showed no significant difference between groups. The GOPEX group showed higher geometric mean pain score on day 7 with fold change 2.29 (95% CI: 1.18–4.44; *P* = 0.015), with no day-specific differences on other days (Fig. 5, Table 3).

**Table 3:** Geometric mean (GM) pain scores, with corresponding 95% confidence intervals for days 1–14 by treatment arm.

Day	GOPEX group, GM (95% CI)	CE group, GM (95% CI)	Fold change (95% CI)	*P* value
**1**	4.41 (3.14–6.20)	3.00 (2.04–4.38)	1.48 (0.98–2.23)	0.065
**2**	1.82 (1.13–2.91)	1.37 (0.82–2.28)	1.33 (0.71–2.49)	0.371
**3**	1.50 (0.93–2.43)	1.10 (0.65–1.85)	1.36 (0.72–2.60)	0.342
**4**	0.91 (0.53–1.57)	0.46 (0.26–0.83)	1.96 (0.94–4.10)	0.073
**5**	0.71 (0.42–1.21)	0.40 (0.23–0.71)	1.78 (0.86–3.65)	0.117
**6**	0.51 (0.30–0.87)	0.29 (0.17–0.52)	1.75 (0.85–3.59)	0.125
**7**	0.41 (0.25–0.68)	0.18 (0.11–0.31)	2.29 (1.18–4.44)	**0**.**015**
**8**	0.23 (0.14–0.36)	0.16 (0.10–0.27)	1.39 (0.74–2.61)	0.296
**9**	0.21 (0.13–0.33)	0.14 (0.09–0.24)	1.46 (0.79–2.70)	0.220
**10**	0.15 (0.10–0.22)	0.13 (0.08–0.20)	1.14 (0.67–1.92)	0.628
**11**	0.12 (0.09–0.18)	0.12 (0.08–0.18)	1.01 (0.63–1.62)	0.964
**12**	0.12 (0.09–0.18)	0.10 (0.07–0.15)	1.23 (0.78–1.93)	0.363
**13**	0.12 (0.08–0.16)	0.10 (0.07–0.15)	1.16 (0.78–1.74)	0.460
**14**	0.10 (0.08–0.14)	0.10 (0.07–0.15)	1.03 (0.72–1.46)	0.882
**Overall**	0.39 (0.28–0.55)	0.28 (0.19–0.41)	1.41 (0.93–2.15)	0.103

Analysed with repeated-measures linear mixed model including operation (GOPEX and CE), day, their interaction, and surgeon as fixed effects. Statistically significant values are shown in bold.

GOPEX, glass ionomer open exposure; CE, closed exposure.

When only the first 7 postoperative days were assessed, estimates of geometric mean pain scores were consistently higher in the GOPEX group than in the CE group and the overall fold change was 1.78 (95% CI: 1.07–2.99; *P* = 0.028), indicating a significant difference between groups. On day 1, the geometric mean pain score was 3.90 (95% CI: 2.64–5.75) in the GOPEX group compared with 2.48 (95% CI: 1.58–3.90) in the CE group, corresponding to a fold change of 1.57 (95% CI: 1.03–2.39; *P* = 0.035). Pain decreased in both groups following a similar pattern, with the difference again reaching significance on day 7 with fold change 2.43 (95% CI: 1.24–4.79; *P* = 0.011) (Supplementary Material S3).

**Figure 6:**
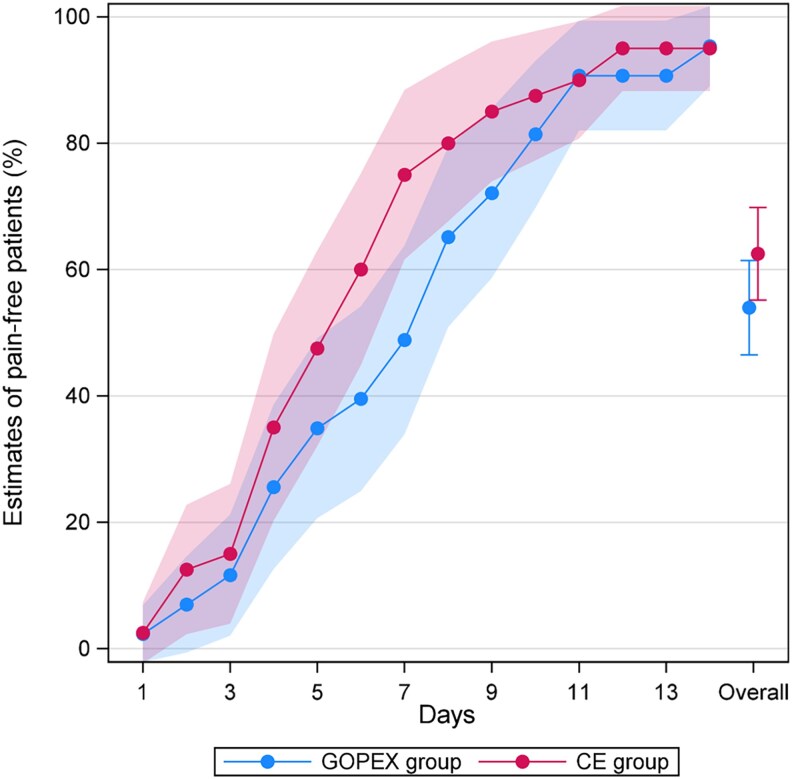
Estimates of the proportion of pain-free patients (pain score 0), with corresponding 95% confidence intervals, for days 1–14 by treatment arm: glass ionomer open exposure (GOPEX) and closed exposure (CE).

Estimated percentages of pain-free patients (pain score 0) over days 1–14 are presented in Table 4 and Fig. 6. The percentage increased steadily in both groups, exceeding 90% by days 11–14. A significant difference was observed on day 7 in favour of the CE group (absolute difference: 26.2%; 95% CI: 6.1, 46.2; *P* = 0.011), with no other day-specific or overall differences (absolute difference: 8.5%; 95% CI: −2.0, 19.0; *P* = 0.111). Similarly, the 1–7-day analysis showed no overall difference between the groups (absolute difference: 11.1%; 95% CI: −1.8, 24.0; *P* = 0.091).

**Table 4:** Percentage of pain-free patients (pain score 0), and estimates of analgesic consumption, each with corresponding 95% confidence intervals for days 1–14 by treatment arm.

Day	Estimate of pain free patients %				Estimate of analgesic consumption %			
	GOPEX group (95% CI)	CE group (95% CI)	Difference % (95% CI)	*P* value	GOPEX group (95% CI)	CE group (95% CI)	Difference % (95% CI)	*P* value
**1**	2.3 (−2.2, 6.8)	2.5 (−2.3, 7.3)	0.2 (−6.4, 6.8)	0.959	90.7 (82.0, 99.4)	92.5 (84.3, 100.7)	1.8 (−10.1, 13.7)	0.767
**2**	7.0 (−0.6, 14.6)	12.5 (2.3, 22.7)	5.5 (−7.2, 18.3)	0.397	88.4 (78.8, 98.0)	80.0 (67.6, 92.4)	−8.4 (−24.0, 7.3)	0.295
**3**	11.6 (2.0, 21.2)	15.0 (3.9, 26.1)	3.4 (−11.3, 18.0)	0.652	72.1 (58.7, 85.5)	60.0 (44.8, 75.2)	−12.1 (−32.3, 8.2)	0.242
**4**	25.6 (12.5, 38.6)	35.0 (20.2, 49.8)	9.4 (−10.3, 29.1)	0.349	67.4 (53.4, 81.4)	40.0 (24.8, 55.2)	−27.4 (−48.1, −6.8)	**0**.**009**
**5**	34.9 (20.6, 49.1)	47.5 (32.0, 63.0)	12.6 (−8.4, 33.6)	0.240	44.2 (29.3, 59.0)	17.5 (5.7, 29.3)	−26.7 (−45.6, −7.7)	**0**.**006**
**6**	39.5 (24.9, 54.1)	60.0 (44.8, 75.2)	20.5 (−0.6, 41.5)	0.057	20.9 (8.8, 33.1)	17.5 (5.7, 29.3)	−3.4 (−20.4, 13.5)	0.691
**7**	48.8 (33.9, 63.8)	75.0 (61.6, 88.4)	26.2 (6.1, 46.2)	**0**.**011**	20.9 (8.8, 33.1)	12.5 (2.3, 22.7)	−8.4 (−24.3, 7.5)	0.299
**8**	65.1 (50.9, 79.4)	80.0 (67.6, 92.4)	14.9 (−4.0, 33.8)	0.122	11.6 (2.0, 21.2)	10.0 (0.7, 19.3)	−1.6 (−15.0, 11.7)	0.811
**9**	72.1 (58.7, 85.5)	85.0 (73.9, 96.1)	12.9 (−4.5, 30.3)	0.146	11.6 (2.0, 21.2)	10.0 (0.7, 19.3)	−1.6 (−15.0, 11.7)	0.811
**10**	81.4 (69.8, 93.0)	87.5 (77.3, 97.7)	6.1 (−9.4, 21.6)	0.440	4.7 (−1.6, 10.9)	5.0 (−1.8, 11.8)	0.3 (−8.9, 9.6)	0.941
**11**	90.7 (82.0, 99.4)	90.0 (80.7, 99.3)	−0.7 (−13.4, 12.0)	0.914	4.7 (−1.6, 10.9)	7.5 (−0.7, 15.7)	2.8 (−7.5, 13.2)	0.588
**12**	90.7 (82.0, 99.4)	95.0 (88.2, 101.8)	4.3 (−6.7, 15.3)	0.443	2.3 (−2.2, 6.8)	2.5 (−2.3, 7.3)	0.2 (−6.4, 6.8)	0.959
**13**	90.7 (82.0, 99.4)	95.0 (88.2, 101.8)	4.3 (−6.7, 15.3)	0.443	2.3 (−2.2, 6.8)	2.5 (−2.3, 7.3)	0.2 (−6.4, 6.8)	0.959
**14**	95.3 (89.1, 101.6)	95.0 (88.2, 101.8)	−0.3 (−9.6, 8.9)	0.941	2.3 (−2.2, 6.8)	2.5 (−2.3, 7.3)	0.2 (−6.4, 6.8)	0.959
**Overall**	54.0 (46.5, 61.5)	62.5 (55.2, 69.8)	8.5 (−2.0, 19.0)	0.111	31.7 (25.8, 37.6)	25.7 (19.4, 32.0)	−6.0 (−14.7, 2.6)	0.173

Analysed using generalised estimating equations (GEE) including operation [glass ionomer open exposure (GOPEX) and CE], day and their interaction as fixed effects. Statistically significant values are shown in bold.

GOPEX, glass ionomer open exposure; CE, closed exposure.

On day 1, patients in both the GOPEX and the CE group reported the most pain before noon (18 and 16 patients, respectively) and in the afternoon (22 and 20 patients, respectively). From day 2 onward, patients in the GOPEX group reported the most pain before noon (21 patients) and continued to do so during the remaining days. In the CE group, the most pain was reported before noon (12 patients) and in the afternoon (13 patients) on day 2, but from day 3 onwards, they also reported the most pain before noon.

In both groups, patients reported that the pain was more intermittent than constant, more gradual than sudden, and more throbbing than stabbing. This pattern persisted throughout the postoperative period for both groups with no differences between the groups (data not shown).

#### Analgesics consumption

**Figure 7:**
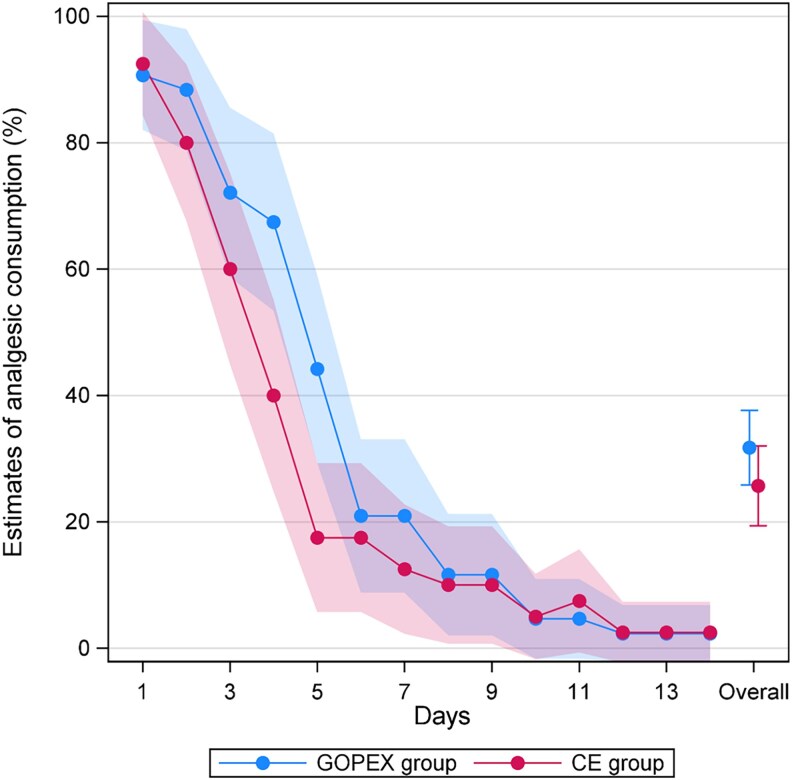
Estimates of analgesic consumption, with corresponding 95% confidence intervals, for days 1–14 stratified by treatment arm: glass ionomer open exposure (GOPEX) and closed exposure (CE).

No patients in the study reported taking any analgesics other than nonsteroidal anti-inflammatory drugs (NSAIDs) and paracetamol. The estimated percentage of patients using analgesics over days 1–14 was not significant between groups overall (absolute difference: −6.0%; 95% CI: −14.7, 2.6; *P* = 0.173). Day-specific analyses showed significantly lower analgesic use in the CE group on postoperative days 4 (absolute difference: −27.4% (95% CI −48.1, −6.8: *P* = 0.009) and 5 (absolute difference: −26.7%; 95% CI: −45.6, −7.7; *P* = 0.006), with no significant differences on other days (Table 4, Fig. 7). Similarly, no other significant difference was observed overall in the 1–7-day analysis.

#### School, leisure activities and disturbed sleep

Supplementary Material S4 summarizes the number of patients in each group who missed school due to the operation, had difficulty with schoolwork, avoided leisure activities, or woke up at night because of pain. Questions were omitted for days when no patients in either group reported the issue. Overall, school absence declined from a majority on the day of surgery to about half the next day, and to only a few patients thereafter. On the first night after the exposure, six patients (14%) in the GOPEX group and five patients (13%) in the CE group woke up at night due to pain. The following night, four patients (9%) in the GOPEX group and two patients (5%) in the CE group experienced this, and by the third night, two patients (5%) in both groups reported waking up due to pain.

#### Bleeding and swelling

On day 1, two patients (5%) in the GOPEX group and three patients (8%) in the CE group reported much or extreme swelling at the surgical site. The number of patients reporting much or extreme bleeding at the surgical site was 3 (7%) in the GOPEX group and 8 (20%) in the CE group on day 1. All days on which any patients reported much, or extreme swelling or bleeding are shown in Supplementary Material S4.

Patients reporting no discomfort on any items were classified as symptom-free. The median time to being symptom-free was nine days in the GOPEX group and six days in the CE group, representing a statistically significant difference (*P* = 0.013).

#### Functional jaw impairment

For the question on jaw impairment both groups reported the greatest difficulties with eating-related tasks, especially chewing hard food. The response option ‘not applicable’ was used when the patient had not tried to perform the specific activity addressed in the question, such as not trying to chew hard food.

The GEE analysis showed that the difference in the percentage of patients reporting difficulty chewing hard food was not significant between groups overall for days 1–14 (absolute difference −5.3; 95% CI: −13.6, 3.0; *P* = 0.212). Day-specific analyses showed that GOPEX group had more difficulties chewing hard food on day 4 (absolute difference: −21.6; 95% CI: −39.5, 3.7; *P* = 0.018) and day 5 (absolute difference: −19.3; 95% CI: −36.1, −2.6; *P* = 0.024) (Supplementary Material S5).

Only a few patients in each group reported experiencing much or extreme difficulty with speaking, taking a big bite, chewing soft food, swallowing, mouth opening, drinking, laughing, or yawning on any postoperative day. In the GOPEX group 28% reported much or extreme difficulties taking a big bite on day 1, and 17% and 12% respectively on day 2 and 3. The corresponding percentages for the CE group were 13% day 1, 15% day 2 and 18% day 3. The jaw function impairment results for days 1–20 by treatment group is presented in Supplementary Material S6. Numbers, percentages, and ‘not applicable’ responses are shown; with ‘not applicable’ responses excluded from percentage calculations. Questions with no reports of notable impairment in any group were omitted for that day.

#### Surgical outcome and duration

A primary tooth was extracted simultaneously with the exposure in 25 patients: 12 in the GOPEX group and 13 in the CE group. All patients, except for four in the GOPEX group and one in the CE group, were sedated with midazolam and/or nitrous oxide. Surgery duration data were missing for 13 of the 83 patients (seven in the GOPEX group and six in the CE group). Day 1 pain scores did not differ significantly between patients with and without missing surgery duration data (*P* = 0.48). Subsequent analyses were conducted using available cases. The median surgery time was 60.0 min in the GOPEX group and 54.5 min in the CE group, showing no statistically significant difference (Table 5).

**Table 5:** Surgical outcome for the two groups: glass ionomer open exposure (GOPEX) and closed exposure (CE).

	GOPEX group (*n* = 43)	CE group (*n* = 40)	*P* value
Extraction of primary tooth	12	13	0.649
Sedation			
No sedation	4	1	
Midazolam	18	21	
Nitrous oxide	1	2	0.503
Midazolam and Nitrous oxide	20	16	
Surgery time (min.) Mean	60.6^a^ SD 22.8	58.5^b^ SD 19.2	
Surgery time (min.) Median	60.0 (Min-Max: 25–120)	54.5 (Min-Max: 35–105)	0.795
Severe complications			
Bleeding	0	0	
Swelling	0	0	
Need for second surgery	0	0	
Infection	0	1	
Minor complications			
Loss of glass ionomer	2^c^	NA	
Chain loose, not from PDC	NA	4	
Total numbers of complications	2	5	0.254

Max, maximum; Min, minimum; NA, not applicable; PDC, Palatallt displaced canine; SD, standard deviation.

^a^Seven patients excluded because of lack of data.

^b^Six patients excluded because of lack of data.

^c^No need for new glass ionomer, one only telephone contact.

#### Complications

Complications reported up to four weeks after the exposure were few in both groups. One patient in the CE group was prescribed antibiotics due to fever and swelling. The total number of complications was not statistically different between the groups (Table 5).

#### Harms

No harmful effects were observed during the study.

## Discussion

The absence of a significant differences in pain, analgesic use, and the proportion of pain-free patients over the entire postoperative period (days 1–14) suggests no major differences between the two intervention groups. However, the analysis of days 1–7 showed a statistically higher geometric mean pain score in the GOPEX group overall for the first week, and specifically on days 1 and 7. By day 7, pain levels were so low in both groups that, despite statistical significance, the difference is unlikely to be clinically relevant. Previous studies have reported greater pain following open surgical exposure [17-19], while others found no differences in pain scores between open and CE [15, 16]. Pain intensity in these studies was assessed at a single time point [15], on postoperative days 1 and 7 [18], or daily for 7 days postoperatively [16, 17, 19]. However, none of these studies have followed patients until they were completely pain-free, limiting the ability to compare the groups across the entire postoperative period. One possible reason why open exposure appears to cause more pain is that this technique leaves a larger wound surface in connection with the GIC compared with CE, where the opening does not need to be larger than what is required for a chain to pass through. Dental fear, which is associated with higher self-reported pain [29], was more frequent in the GOPEX group (four vs one patient above the CFSS-DC clinical threshold) [25, 26]. Although this could have affected postoperative pain, the difference was not statistically significant (*P* = 0.07) and is unlikely to have had a meaningful impact.

The GEE analysis of proportion of pain-free patients over days 1–14 showed no overall statistically significant difference, although a statistically significant was observed on day 7. Additionally, patients in the GOPEX group took significantly longer to report being completely symptom-free, indicating a slightly longer recovery period. This finding aligns with other reporting extended recovery times following open exposure [16, 17, 19]. Two patients reported pain up to postoperative day 19, and overall, 27% of patients across both groups still experienced pain after day 7. Although mean pain levels were low after day 7, spaghetti plots show that some patients had clinically significant pain beyond this point, a finding not reported in previous studies. Of the two patients with the longest pain duration, one was in the GOPEX group (PDC in sector 5 with the second-longest cusp–occlusal plane distance) and the other in the CE group who developed a postoperative infection, likely explaining their prolonged pain.

Already on day 1, patients described their pain as more intermittent than constant, a pattern that persisted throughout the postoperative period. This may be explained by the influence of daily activities on pain perception, such as increased pain during meals compared with rest. Additionally, pain fluctuations could be attributed to the timing and frequency of analgesic intake. All patients were advised to take analgesics every 4–6 h for up to 5 days; however, not everyone adhered to this recommendation, which may have contributed to the intermittent nature of the pain.

Analgesic intake followed the same trend in both groups, with the GEE analysis showing no overall difference for days 1–14. However, day-specific analysis on days 4 and 5 showed a higher proportion of GOPEX patients taking analgesics. These days also corresponded to when they experience more difficulty chewing hard food. The higher analgesic consumption in the GOPEX group aligns with the findings of Chaushu *et al*. [17] who reported greater analgesic use in the open exposure group on days 3 to 5 but does not correspond to the findings of Fernandes Færøvig *et al*. [19] reporting no differences between groups. Analgesic consumption cannot be compared with the studies of Gharabei *et al*. [16] where free analgesics were provided, Björksved *et al*. [18] where no data were collected for day 2–6, and Parkin *et al*. [15] where data were only collected once.

Gharaibeh *et al*. [16] and Fernandes Færøvig *et al*. [19] found that the duration of the surgery was significantly shorter in the open exposure group, measured from the initial incision to the final suture. However, the inverse outcome was found by Chaushu *et al*. [17], with significantly shorter surgical duration in the closed group. The lack of congruent conclusions is demonstrated further by Parkin *et al*. [15] and Björksved *et al*. [18] who found no difference between open and closed surgical durations where the former used a surgical dressing, and the latter used GIC. The present study found no differences in surgery duration between the groups.

Variations in how complications are reported make it difficult to compare these issues across studies. Björksved *et al*. [18] reported complications up to 4 weeks postsurgery and defined it as a condition in need of a dental care visit. Infection was defined as the need for antibiotics. Fernandes Færøvig *et al*. [19] reported complications up to 3 weeks postsurgery, which included clinician-reported complications such as bleeding, swelling, infection, loss of suture, bonding failure and loss of dressing. Parkin *et al*. [15] reported ‘failure rate’ defined as need of reoperation and ‘other complications’ that also included the orthodontic treatment. In the present study, no significant differences were found between the groups and the total complication rate was 4.7% in the GOPEX group and 12.5% in the CE group. Unlike Björksved *et al*. [18] and Fernandes Færøvig *et al*. [19] our study did not group ‘gingival overgrowth’ and ‘loss of GIC’ together since gingival overgrowth require reoperation, but only loss of GIC may not require an intervention. However, to compare our results with the previous study, we made the same categorization. Therefore, in our study the complication rate for gingival overgrowth/loss of GIC in the GOPEX group was 4.7%. In the CE group, 10.0% had loss of chain from the adjacent tooth, not the exposed canine. The corresponding figures in the study of Björksved *et al*. [18] was a total complication rate of 20.3% in the open group and 21.7% in the closed group; 6.8% gingival overgrowth/loss of GIC and 15.0% loss of chain. In contrast to our study, they also found a significant difference between groups with more severe complications in the open group, a significance that was no longer present when bilateral cases were excluded. In both our study and the one conducted by Björksved *et al*. [18], GIC was used for the open exposure. However, a key methodological difference is that in our study, GIC was applied after the mucoperiosteal flap was sutured back, while in their study, it was placed before suturing. Fernandes Færøvig *et al*. [19] used a surgical dressing that was sutured in the open exposure group and reported a 26.7% complication rate in the open exposure group and 10.6% in the CE group, indicating a weak significant difference between the two groups. Bleeding was the most common complication, affecting 17.8% of patients in the open group, compared with none in our study. Loss of dressing or gingival overgrowth occurred in 8.9% of patients in the open exposure group and loss of chain occurred in 8.5% of the patients in the CE group. One patient in the current study developed a postoperative infection (CE group) which is comparable to other studies [15, 18, 19].

### Strengths and limitations

Daily comparisons were performed for descriptive purposes, and *P*-values were not adjusted for multiple testing. Therefore, the day-specific results should be interpreted cautiously.

Blinding of the paediatric dentist performing the exposures was not possible due to the study design. The sample size calculation was based solely on differences in pain scores, so results for other outcomes remain uncertain, and a larger sample could have influenced their statistical significance. While telephone collection of patient-reported data has advantages, it also has limitations. Patients may have had less opportunity to reflect on their responses compared with a written format. Another limitation is that the study design did not allow a detailed assessment of how analgesic use influenced reported pain. Patients were not asked about the frequency of medication intake or whether it was taken preventively or in response to pain. Although all patients received standardised instructions to take paracetamol and ibuprofen every 4–6 h for the first 5 days, it is unclear who followed this schedule regardless of symptoms and who took medication only when experiencing pain.

The strengths of this study include its exclusive focus on patients with unilateral PDCs treated under local anaesthesia and the collection of patient-reported data daily until patients were symptom-free, facilitating a comprehensive day-by-day analysis of postoperative experience. Data were collected via telephone interviews, allowing the patients to ask for clarification, reducing recall bias, and minimising nonresponse or patients reporting for multiple days at once. Patients with planned premolar extractions were excluded, as this could have influenced postoperative pain. Importantly, PDCs of all displacement severities, including sector 5, were included, demonstrating that GOPEX can be applied even when the PDC is close to the midpalate suture assessed on panoramic radiographs.

### Generalisability

The results can be generalised to patients <18 years of age with a PDC in sectors 2–5 requiring surgical exposure. However, according to a recently published article [14], GOPEX should be avoided in cases where the PDC is too close to an adjacent tooth and the GIC filling may affect the root of that tooth, or when it is positioned so deeply that the GIC filling would need to be excessively large. Procedures were performed by three surgeons, each with at least ten years of experience in both techniques, enhancing the generalisability of the findings. Although not a multicentre study, multiple operators provide comparable or greater generalisability than some multicentre studies [18, 19].

### Clinical implications and future research

The results of this study can be used to inform patients about what to expect following an exposure procedure. However, it is not possible to provide a definitive prediction of postoperative pain levels or the time to being pain-free. Patients in both groups reported a wide range of experiences, from ‘no pain at all’ to ‘worst imaginable pain’. This wide individual variation likely reflects both actual differences in pain intensity and subjective factors, including each patient’s prior pain experiences and personal pain tolerance. Moderate pain can be expected for the first 2–3 days, followed by mild pain for an additional 3–5 days. Most patients are pain-free after 1 week, though considerable individual variation exists. The GOPEX group seems to have a longer recovery time and more pain the first postoperative week. Few patients experienced significant swelling or bleeding, school and leisure activities were affected for most patients only on the day following the exposure, and complications were rare and generally uncomplicated.

Not only factors related to the surgical exposure itself are important when selecting between open or CE. The time required for the PDC to erupt, the duration and success of orthodontic treatment, and post-treatment outcomes, such as periodontal and aesthetic differences between the methods, have previously been investigated [30-34]. Ongoing research is also addressing these questions, along with pain during orthodontic traction, with results anticipated to be published soon.

## Conclusions

- Considerable individual variation was seen for all outcomes and in both groups.
- The GOPEX group had more pain the first postoperative week, but no difference was seen in pain score or analgesic consumption overall for the whole postoperative period.
- There were no differences between groups regarding surgery duration, complications or impact on daily life.

## Supplementary Material

cjag011_Supplementary_Data

## Data Availability

Data underlying this publication will be shared on reasonable request to the corresponding author.
