# Correction to: Glass ionomer open exposure and closed exposure of palatally displaced canines: a randomised controlled trial comparing postoperative pain perception and complications

**DOI:** 10.1093/ejo/cjag052

**Published:** 2026-06-17

This is a correction to: Anna Dahlén, Viktoria Tagesson, Damon Taheri, Ken Hansen, Larisa Krekmanova, Julia Naoumova, Glass ionomer open exposure and closed exposure of palatally displaced canines: a randomised controlled trial comparing postoperative pain perception and complications, *European Journal of Orthodontics*, Volume 48, Issue 2, April 2026, cjag011, https://doi.org/10.1093/ejo/cjag011

In the originally published version of this manuscript, a typographical error was present in the title. The word “trial” was inadvertently misspelled as “trail.”

This has now been corrected.

