# Supplementary material for: Glass ionomer open exposure and closed exposure of palatally displaced canines: a randomised controlled trial comparing postoperative pain perception and complications
Source: Eur J Orthod. 2026 Mar 17;48(2):cjag011. doi: 10.1093/ejo/cjag011 (PMC13016904; doi:10.1093/ejo/cjag011)
Supplement: cjag011_Supplementary_Data [file cjag011_supplementary_data.zip › Supplementary Material 1.docx]

**Supplementary Material 1**.
Dental Subscale Children’s Fear Schedule Survey (CFSS-DS)

**Try to estimate how afraid you are in the following situations.**
Mark your answer with an **X** in the appropriate box. Only one answer per question. Please answer all questions.

**How afraid are you…**

| **Situation** | **Not at all afraid** | **Only a little afraid** | **Quite afraid** | **Very afraid** | **Extremely afraid** |
| --- | --- | --- | --- | --- | --- |
| Of the dentist | ( ) | ( ) | ( ) | ( ) | ( ) |
| Of the doctor | ( ) | ( ) | ( ) | ( ) | ( ) |
| Of getting an injection or anaesthesia | ( ) | ( ) | ( ) | ( ) | ( ) |
| When someone examines your teeth/mouth | ( ) | ( ) | ( ) | ( ) | ( ) |
| When you open your mouth at the dentist | ( ) | ( ) | ( ) | ( ) | ( ) |
| When a stranger touches you | ( ) | ( ) | ( ) | ( ) | ( ) |
| When somebody looks at you | ( ) | ( ) | ( ) | ( ) | ( ) |
| When the dentist drills in your tooth | ( ) | ( ) | ( ) | ( ) | ( ) |
| The sight of the dentist drilling | ( ) | ( ) | ( ) | ( ) | ( ) |
| The noise of the dentist drilling | ( ) | ( ) | ( ) | ( ) | ( ) |
| When someone puts instruments in your mouth | ( ) | ( ) | ( ) | ( ) | ( ) |
| Of choking | ( ) | ( ) | ( ) | ( ) | ( ) |
| Of needing to go to the hospital | ( ) | ( ) | ( ) | ( ) | ( ) |
| Of people in white hospital or dental uniforms | ( ) | ( ) | ( ) | ( ) | ( ) |
| When someone cleans or applies fluoride to your teeth | ( ) | ( ) | ( ) | ( ) | ( ) |
