# Supplementary material for: Glass ionomer open exposure and closed exposure of palatally displaced canines: a randomised controlled trial comparing postoperative pain perception and complications
Source: Eur J Orthod. 2026 Mar 17;48(2):cjag011. doi: 10.1093/ejo/cjag011 (PMC13016904; doi:10.1093/ejo/cjag011)
Supplement: cjag011_Supplementary_Data [file cjag011_supplementary_data.zip › Supplementary Material 2.docx]

**Supplementary Material 2: Questionnaire**

Questionnaire answered every day starting on the evening of the surgery (day 1) and continuing until patient reported being pain-free, symptom-free, and no longer using analgesics.

| Questions | Respons alternative |
| --- | --- |
| 1. **Did you find any part of the operation particularly unpleasant?**  If yes, what....................................................... | Yes/no |
| 1. **How much pain did you have today, when it was the most intense, on a scale from 0-10? Zero indicated "no pain at all" and 10 indicated the "worst pain imaginable."** | 0-10 |
| 1. **What time of day has it been most pronounced?** | Night/before noon /afternoon/evening |
| 1. **How can you best describe the pain you have?** | -Constant/intermittent  -Sudden/gradual  -Throbbing/stabbing |
| 1. **Have you taken analgesics for the pain today?** If yes, what.............................. | Yes/no |
| 1. **How much difficulties have you had today, because of the surgical exposure, regarding**: -Speech -Take av big bite -Chew hard food -Chew soft food -Swallow -Open your mouth -Do schoolwork -Drink -Laugh   -Yawn | not at all/slightly difficult/very difficult/extremely difficult/not applicable |
| 1. **Have you today in the surgical area had**: -Bleeding -Swelling | Not at all/slight/much/ extreme |
| 1. **Have you been absent from school because of pain after the surgical exposure?** | Yes/no/not applicable |
| 1. **Have you been absent from leisure activities due to pain after the operation**? If yes, what....................................................... | Yes/no/not applicable |
| 1. **Have you woken up at night because of pain after the surgical exposure?** (to be answered from day 2) | Yes/no |
