# Supplementary material for: Glass ionomer open exposure and closed exposure of palatally displaced canines: a randomised controlled trial comparing postoperative pain perception and complications
Source: Eur J Orthod. 2026 Mar 17;48(2):cjag011. doi: 10.1093/ejo/cjag011 (PMC13016904; doi:10.1093/ejo/cjag011)
Supplement: cjag011_Supplementary_Data [file cjag011_supplementary_data.zip › Supplementary Material 3.docx]

**Supplementary Material 3**


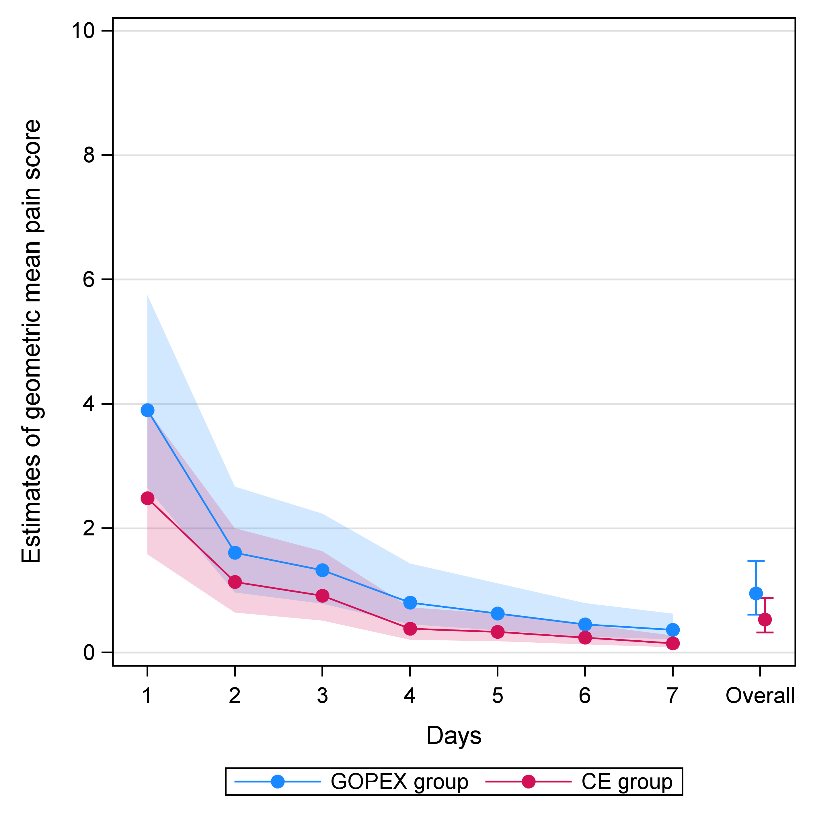
Geometric mean (GM) pain scores with corresponding 95% confidence intervals for days 1–7, stratified by treatment arm.

| **Day** | **Gopex group, GM (95% CI)** | **CE group, GM (95% CI)** | **Fold change (95% CI)** | **P value** |
| --- | --- | --- | --- | --- |
| **1** | 3.90 (2.64-5.75) | 2.48 (1.58-3.90) | 1.57 (1.03-2.39) | **0.035** |
| **2** | 1.60 (0.96-2.67) | 1.13 (0.64-2.00) | 1.41 (0.75-2.67) | 0.282 |
| **3** | 1.32 (0.79-2.23) | 0.91 (0.51-1.63) | 1.45 (0.75-2.80) | 0.264 |
| **4** | 0.81 (0.45-1.43) | 0.39 (0.21-0.73) | 2.09 (0.99-4.40) | 0.053 |
| **5** | 0.63 (0.36-1.11) | 0.33 (0.18-0.62) | 1.89 (0.91-3.93) | 0.087 |
| **6** | 0.45 (0.26-0.80) | 0.24 (0.12-0.45) | 1.86 (0.90-3.87) | 0.093 |
| **7** | 0.37 (0.21-0.62) | 0.15 (0.08-0.27) | 2.43 (1.24-4.79) | **0.011** |
| **Overall** | 0.95 (0.61-1.47) | 0.53 (0.32-0.88) | 1.78 (1.07-2.99) | **0.028** |

Note. Analysed using repeated-measures linear mixed model including operation (glass ionomer open exposure (GOPEX) and closed exposure (CE)), day, their interaction, and surgeon as fixed effects.
