# Supplementary material for: Glass ionomer open exposure and closed exposure of palatally displaced canines: a randomised controlled trial comparing postoperative pain perception and complications
Source: Eur J Orthod. 2026 Mar 17;48(2):cjag011. doi: 10.1093/ejo/cjag011 (PMC13016904; doi:10.1093/ejo/cjag011)
Supplement: cjag011_Supplementary_Data [file cjag011_supplementary_data.zip › Supplementary Material 4.docx]

**Supplementary Material 4**Patient-reported outcomes including absence from school, much or extreme difficulty with schoolwork, refraining from leisure activities, presence of significant swelling or bleeding at the surgical site, or awakening at night due to pain.

|  | GOPEX group  (n=43) | | | CE group  (n=40) | | |
| --- | --- | --- | --- | --- | --- | --- |
|  | n | % | n/a | n | % | n/a |
| Day 1 |  |  |  |  |  |  |
| Absent school | 34 | 89 | 5 | 30 | 81 | 3 |
| Difficulties to do schoolwork | 0 | 0 | 30 | 1 | 9 | 29 |
| Refrained from leisure activities | 20 | 56 | 7 | 14 | 42 | 7 |
| Much/extreme swelling | 2 | 5 | NA | 3 | 8 | NA |
| Much/extreme bleeding | 3 | 7 | NA | 8 | 20 | NA |
| Day 2 |  |  |  |  |  |  |
| Absent school | 16 | 57 | 15 | 12 | 40 | 10 |
| Difficulties to do schoolwork | 0 | 0 | 21 | 1 | 3 | 9 |
| Refrained from leisure activities | 11 | 33 | 8 | 10 | 29 | 5 |
| Woken up at night | 6 | 14 | NA | 5 | 13 | NA |
| Much/extreme swelling | 7 | 16 | NA | 3 | 8 | NA |
| Much/extreme bleeding | 0 | 0 | NA | 2 | 5 | NA |
| Day 3 |  |  |  |  |  |  |
| Absent school | 4 | 18 | 21 | 2 | 8 | 14 |
| Refrained from leisure activities | 6 | 17 | 7 | 4 | 11 | 5 |
| Woken up at night | 4 | 9 | NA | 2 | 5 | NA |
| Much/extreme swelling | 3 | 7 | NA | 2 | 5 | NA |
| Day 4 |  |  |  |  |  |  |
| Absent school | 0 | 0 | 19 | 1 | 4 | 14 |
| Difficulties to do schoolwork | 0 | 0 | 10 | 2 | 6 | 7 |
| Refrained from leisure activities | 3 | 8 | 4 | 2 | 5 | 3 |
| Woken up at night | 2 | 5 | NA | 2 | 5 | NA |
| Much/extreme swelling | 0 | 0 | NA | 2 | 5 | NA |
| Day 5 |  |  |  |  |  |  |
| Refrained from leisure activities | 2 | 6 | 7 | 1 | 8 | 3 |
| Woken up at night | 1 | 2 | NA | 1 | 3 | NA |
| Much/extreme swelling | 1 | 2 | NA | 2 | 5 | NA |
| Day 6 |  |  |  |  |  |  |
| Absent school | 1 | 3 | 12 | 1 | 4 | 12 |
| Refrained from leisure activities | 1 | 3 | 3 | 1 | 3 | 3 |
| Woken up at night | 2 | 5 | NA | 2 | 5 | NA |
| Much/extreme swelling | 0 | 0 | NA | 1 | 3 | NA |
| Much/extreme bleeding | 0 | 0 | NA | 1 | 3 | NA |
| Day 7 |  |  |  |  |  |  |
| Absent school | 1 | 3 | 6 | 2 | 5 | 1 |
| Refrained from leisure activities | 1 | 3 | 3 | 1 | 3 | 4 |
| Woken up at night | 1 | 2 | NA | 1 | 3 | NA |
| Much/extreme swelling | 1 | 2 | NA | 0 | 0 | NA |
| Much/extreme bleeding | 0 | 0 | NA | 1 | 3 | NA |
| Day 12 |  |  |  |  |  |  |
| Much/extreme swelling | 1 | 2 | NA | 0 | 0 | NA |
| Much/extreme bleeding | 1 | 2 | NA | 0 | 0 | NA |

Note. n, number of patients; %, percentage calculations without not applicable responses; NA, not applicable. Questions were omitted for days when no patients in either group reported the issue.
