# Supplementary material for: Glass ionomer open exposure and closed exposure of palatally displaced canines: a randomised controlled trial comparing postoperative pain perception and complications
Source: Eur J Orthod. 2026 Mar 17;48(2):cjag011. doi: 10.1093/ejo/cjag011 (PMC13016904; doi:10.1093/ejo/cjag011)
Supplement: cjag011_Supplementary_Data [file cjag011_supplementary_data.zip › Supplementary Material 5.docx]

**Supplementary Material 5**


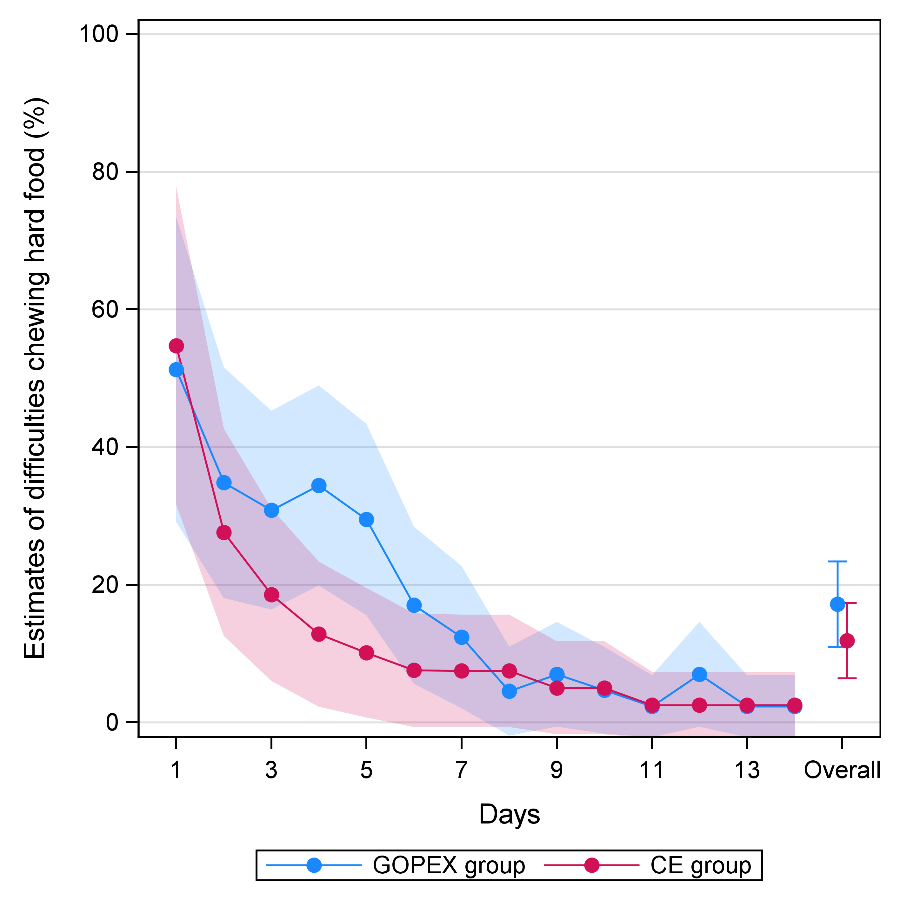
 Estimated proportions of patients reporting severe or extreme difficulty chewing hard food, with corresponding 95% confidence intervals, for days 1–14 by treatment arm.

| **Day** | **GOPEX group % (95% CI)** | **CE group % (95% CI)** | **Difference % (95% CI)** | **P value** |
| --- | --- | --- | --- | --- |
| 1 | 51.3 (29.1, 73.4) | 54.7 (31.7, 77.8) | 3.4 (-28.5, 35.4) | 0.833 |
| 2 | 34.8 (18.0, 51.6) | 27.6 (12.5, 42.6) | -7.2 (-29.8, 15.3) | 0.530 |
| 3 | 30.8 (16.4, 45.2) | 18.5 (6.0, 31.1) | -12.3 (-31.4, 6.8) | 0.208 |
| 4 | 34.4 (19.9, 48.9) | 12.8 (2.3, 23.3) | -21.6 (-39.5, -3.7) | **0.018** |
| 5 | 29.5 (15.6, 43.4) | 10.1 (0.7, 19.5) | -19.3 (-36.1, -2.6) | **0.024** |
| 6 | 17.0 (5.6, 28.4) | 7.6 (-0.7, 15.8) | -9.4 (-23.5, 4.7) | 0.190 |
| 7 | 12.4 (2.1, 22.7) | 7.5 (-0.7, 15.7) | -4.9 (-18.0, 8.3) | 0.468 |
| 8 | 4.5 (-1.9, 11.0) | 7.5 (-0.7, 15.7) | 3.0 (-7.4, 13.4) | 0.575 |
| 9 | 7.0 (-0.6, 14.6) | 5.0 (-1.8, 11.8) | -2.0 (-12.2, 8.2) | 0.704 |
| 10 | 4.7 (-1.6, 10.9) | 5.0 (-1.8, 11.8) | 0.3 (-8.9, 9.6) | 0.941 |
| 11 | 2.3 (-2.2, 6.8) | 2.5 (-2.3, 7.3) | 0.2 (-6.4, 6.8) | 0.959 |
| 12 | 7.0 (-0.6, 14.6) | 2.5 (-2.3, 7.3) | -4.5 (-13.5, 4.5) | 0.331 |
| 13 | 2.3 (-2.2, 6.8) | 2.5 (-2.3, 7.3) | 0.2 (-6.4, 6.8) | 0.959 |
| 14 | 2.3 (-2.2, 6.8) | 2.5 (-2.3, 7.3) | 0.2 (-6.4, 6.8) | 0.959 |
| Overall | 17.2 (10.9, 23.4) | 11.9 (6.4, 17.3) | -5.3 (-13.6, 3.0) | 0.212 |

Note. Analysed using generalized estimating equations (GEE) including operation (glass ionomer open exposure (GOPEX) and closed exposure (CE)), day and their interaction as fixed effects.
