# Supplementary material for: Glass ionomer open exposure and closed exposure of palatally displaced canines: a randomised controlled trial comparing postoperative pain perception and complications
Source: Eur J Orthod. 2026 Mar 17;48(2):cjag011. doi: 10.1093/ejo/cjag011 (PMC13016904; doi:10.1093/ejo/cjag011)
Supplement: cjag011_Supplementary_Data [file cjag011_supplementary_data.zip › Supplementary Material 6.docx]

**Supplementary Material 6**

Patient reported much or extreme difficulty to speak, take a big, chew hard and soft food, swallow, open the mouth, drink, laugh and yawn due to pain from the surgery.

|  | GOPEX group  (n=43) | | | CE group  (n=40) | | |
| --- | --- | --- | --- | --- | --- | --- |
|  | n | % | n/a | n | % | n/a |
| Day 1 |  |  |  |  |  |  |
| Speak | 3 | 7 | 0 | 3 | 8 | 0 |
| Take a big bite | 9 | 28 | 11 | 4 | 13 | 8 |
| Chew hard food | 10 | 59 | 26 | 11 | 69 | 24 |
| Chew soft food | 4 | 12 | 10 | 3 | 9 | 8 |
| Swallow | 1 | 2 | 0 | 0 | 0 | 0 |
| Open your mouth | 0 | 0 | 0 | 1 | 3 | 0 |
| Drink | 0 | 0 | 1 | 0 | 0 | 1 |
| Laugh | 1 | 2 | 3 | 1 | 3 | 3 |
| Day 2 |  |  |  |  |  |  |
| Speak | 3 | 7 | 0 | 2 | 5 | 0 |
| Take a big bite | 6 | 17 | 8 | 6 | 15 | 1 |
| Chew hard food | 11 | 38 | 14 | 9 | 30 | 10 |
| Chew soft food | 4 | 9 | 0 | 1 | 3 | 1 |
| Swallow | 1 | 2 | 0 | 2 | 5 | 0 |
| Drink | 1 | 2 | 0 | 2 | 5 | 0 |
| Yawn | 0 | 0 | 1 | 2 | 5 | 1 |
| Day 3 |  |  |  |  |  |  |
| Speak | 1 | 2 | 0 | 1 | 3 | 0 |
| Take a big bite | 5 | 12 | 1 | 7 | 18 | 1 |
| Chew hard food | 12 | 32 | 6 | 7 | 19 | 4 |
| Chew soft food | 2 | 5 | 0 | 0 | 0 | 0 |
| Drink | 0 | 0 | 0 | 1 | 3 | 0 |
| Day 4 |  |  |  |  |  |  |
| Speak | 0 | 0 | 0 | 2 | 5 | 0 |
| Take a big bite | 3 | 7 | 2 | 3 | 8 | 0 |
| Chew hard food | 14 | 35 | 3 | 5 | 13 | 2 |
| Chew soft food | 1 | 2 | 0 | 2 | 5 | 0 |
| Swallow | 1 | 2 | 0 | 1 | 3 | 0 |
| Drink | 0 | 0 | 0 | 2 | 5 | 0 |
| Day 5 |  |  |  |  |  |  |
| Take a big bite | 3 | 7 | 1 | 1 | 3 | 0 |
| Chew hard food | 12 | 30 | 3 | 4 | 10 | 1 |
| Chew soft food | 3 | 7 | 0 | 2 | 5 | 0 |
| Swallow | 1 | 2 | 0 | 1 | 3 | 0 |
| Day 6 |  |  |  |  |  |  |
| Take a big bite | 1 | 2 | 1 | 1 | 3 | 0 |
| Chew hard food | 7 | 17 | 2 | 3 | 8 | 1 |
| Chew soft food | 3 | 7 | 0 | 2 | 5 | 0 |
| Swallow | 0 | 0 | 0 | 1 | 3 | 0 |
| Day 7 |  |  |  |  |  |  |
| Take a big bite | 1 | 2 | 1 | 1 | 3 | 0 |
| Chew hard food | 4 | 10 | 2 | 3 | 8 | 0 |
| Chew soft food | 2 | 5 | 1 | 2 | 5 | 0 |
| Open your mouth | 1 | 2 | 0 | 0 | 0 | 0 |
| Drink | 1 | 2 | 0 | 0 | 0 | 0 |
| Laugh | 1 | 2 | 0 | 0 | 0 | 0 |
| Day 8 |  |  |  |  |  |  |
| Take a big bite | 0 | 0 | 0 | 1 | 3 | 0 |
| Chew hard food | 2 | 5 | 0 | 3 | 8 | 0 |
| Chew soft food | 1 | 2 | 0 | 1 | 3 | 0 |
| Day 9 |  |  |  |  |  |  |
| Take a big bite | 2 | 5 | 0 | 0 | 0 | 0 |
| Chew hard food | 3 | 7 | 0 | 2 | 5 | 0 |
| Chew soft food | 1 | 2 | 0 | 1 | 3 | 0 |
| Swallow | 1 | 2 | 0 | 0 | 0 | 0 |
| Day 10 |  |  |  |  |  |  |
| Take a big bite | 1 | 2 | 0 | 0 | 0 | 0 |
| Chew hard food | 2 | 5 | 0 | 2 | 5 | 0 |
| Chew soft food | 0 | 0 | 0 | 1 | 3 | 0 |
| Day 11 |  |  |  |  |  |  |
| Take a big bite | 1 | 2 | 0 | 0 | 0 | 0 |
| Chew hard food | 2 | 5 | 0 | 0 | 0 | 0 |
| Chew soft food | 1 | 2 | 0 | 0 | 0 | 0 |
| Day 12 |  |  |  |  |  |  |
| Take a big bite | 2 | 5 | 0 | 0 | 0 | 0 |
| Chew hard food | 3 | 7 | 0 | 1 | 3 | 0 |
| Chew soft food | 1 | 2 | 0 | 0 | 0 | 0 |
| Swallow | 1 | 2 | 0 | 0 | 0 | 0 |
| Day 13 |  |  |  |  |  |  |
| Take a big bite | 1 | 2 | 0 | 0 | 0 | 0 |
| Chew hard food | 1 | 2 | 0 | 1 | 3 | 0 |
| Day 14 |  |  |  |  |  |  |
| Take a big bite | 1 | 2 | 0 | 0 | 0 | 0 |
| Chew hard food | 1 | 2 | 0 | 1 | 3 | 0 |
| Day 15 |  |  |  |  |  |  |
| Take a big bite | 1 | 2 | 0 | 0 | 0 | 0 |
| Chew hard food | 1 | 2 | 0 | 1 | 3 | 0 |
| Day 16 |  |  |  |  |  |  |
| Take a big bite | 1 | 2 | 0 | 0 | 0 | 0 |
| Chew hard food | 0 | 0 | 0 | 1 | 3 | 0 |
| Day 17 |  |  |  |  |  |  |
| Take a big bite | 1 | 2 | 0 | 0 | 0 | 0 |
| Chew hard food | 2 | 5 | 0 | 0 | 0 | 0 |
| Chew soft food | 1 | 2 | 0 | 0 | 0 | 0 |
| Swallow | 1 | 2 | 0 | 0 | 0 | 0 |
| Day 18 |  |  |  |  |  |  |
| Chew hard food | 1 | 2 | 0 | 0 | 0 | 0 |

Note. N, number of patients; %, percentage calculations without not applicable responses; NA, not applicable. Questions were omitted for days when no patients in either group reported the issue.
